# Blood-Based Next-Generation Sequencing in Adrenocortical Carcinoma

**DOI:** 10.1093/oncolo/oyac061

**Published:** 2022-04-24

**Authors:** Bassel Nazha, Tony Z Zhuang, Hiba I Dada, Leylah M Drusbosky, Jacqueline T Brown, Deepak Ravindranathan, Bradley C Carthon, Omer Kucuk, Jamie Goldman, Viraj A Master, Mehmet Asim Bilen

**Affiliations:** Winship Cancer Institute of Emory University, Atlanta, GA, USA; Department of Hematology and Medical Oncology, Emory University School of Medicine, Atlanta, GA, USA; Department of Medicine, Emory University School of Medicine, Atlanta, GA, USA; Guardant Health, Redwood City, CA, USA; Guardant Health, Redwood City, CA, USA; Winship Cancer Institute of Emory University, Atlanta, GA, USA; Department of Hematology and Medical Oncology, Emory University School of Medicine, Atlanta, GA, USA; Winship Cancer Institute of Emory University, Atlanta, GA, USA; Department of Hematology and Medical Oncology, Emory University School of Medicine, Atlanta, GA, USA; Winship Cancer Institute of Emory University, Atlanta, GA, USA; Department of Hematology and Medical Oncology, Emory University School of Medicine, Atlanta, GA, USA; Winship Cancer Institute of Emory University, Atlanta, GA, USA; Department of Hematology and Medical Oncology, Emory University School of Medicine, Atlanta, GA, USA; Winship Cancer Institute of Emory University, Atlanta, GA, USA; Department of Hematology and Medical Oncology, Emory University School of Medicine, Atlanta, GA, USA; Winship Cancer Institute of Emory University, Atlanta, GA, USA; Department of Urology, Emory University School of Medicine, Atlanta, GA, USA; Winship Cancer Institute of Emory University, Atlanta, GA, USA; Department of Hematology and Medical Oncology, Emory University School of Medicine, Atlanta, GA, USA

**Keywords:** adrenocortical carcinoma, adrenal cancer, next-generation sequencing, circulating tumor DNA (ctDNA), personalized medicine

## Abstract

**Background:**

Adrenocortical carcinoma (ACC) is a rare and heterogeneous malignancy with poor prognosis. We aimed to evaluate the feasibility of next-generation sequencing (NGS) testing of circulating cell-free tumor DNA (ctDNA) in patients with ACC, to characterize the genomic landscape of alterations, and to identify potential clinically actionable mutations.

**Methods:**

Retrospective analysis of genomic data from 120 patients with ACC who had ctDNA testing between 12/2016 and 10/2021 using Guardant360 (Guardant Health, CA) was performed. ctDNA NGS analysis interrogated single nucleotide variants, fusions, indels, and copy number amplifications of up to 83 genes. The frequency of genomic alterations, landscape of co-occurring mutations, and pathogenic/likely pathogenic alterations with potential targeted therapies was identified. The prevalence of alterations identified in ctDNA was compared to those detected in tissue using a publicly available database (cBioPortal).

**Results:**

The median age of this cohort was 53 years (range 21-81), and 56% of patients were female. Ninety-six patients (80%) had ≥1 somatic alteration detected. *TP53* (52%), *EGFR* (23%), *CTNNB1* (18%), *MET* (18%), and *ATM* (14%) were found to be the most frequently altered genes in ACC samples. Pathogenic and/or likely pathogenic mutations in therapeutically relevant genes were observed in 56 patients (47%) and included *EGFR*, *BRAF*, *MET*, *CDKN2A*, *CDK4/6*, and *ATM*. The most frequent co-occurring mutations were *EGFR* + *MET* (9%), *MET* + CDK4 (7%), *EGFR* + *CDK4* (7%), and *BRAF* + *MET* (7%). The frequencies of mutations detected in ctDNA were similar to those detected in tissue.

**Conclusions:**

Utilizing blood-based NGS to characterize genomic alterations in advanced ACC is feasible in over 80% of patients. Almost half of the patients had actionable mutations with approved therapies in other cancers. This approach might inform the development of personalized treatment options or identify clinical trials available for this aggressive malignancy.

## Teaser

Developing a comprehensive understanding of the genomic landscape of adrenocortical carcinoma using ctDNA is an unmet need that may pave the way to incorporating target-based therapies. This article presents the largest study to date evaluating the feasibility of a blood-based ctDNA approach in revealing clinically significant alterations and co-existing genomic alterations in adrenocortical carcinoma.

**Implications for Practice**

Adrenocortical carcinoma (ACC) is a rare and aggressive malignancy with limited treatment options beyond conventional chemotherapy in the advanced or metastatic setting. We show that a blood-based next-generation sequencing was able to detect circulating tumor DNA in over 80% of patients and revealed potentially targetable mutations. Our findings indicate that the use of this assay is feasible in clinical practice and might inform the development of personalized treatment approaches or enrollment in molecularly selected clinical trials for patients with ACC.

## Introduction

Adrenocortical carcinoma (ACC) is a rare malignancy with an incidence of 1-2 per million persons and poor prognosis in the advanced setting.^1^ Among adults, the neoplasm is often seen in individuals aged 40-60 years old with a median age of 55 years at diagnosis. While most cases are sporadic, ACC can be seen in the context of hereditary syndromes, including Li-Fraumeni, Beckwith-Wiedemann, and multiple endocrine neoplasia.^2,3^ Around half of the patients present with acute hypercortisolism or hyperandrogenism.^4^ Over half of patients have Stage III or Stage IV at diagnosis, which has a poor prognosis.^5^ Surgical resection is the gold standard for the treatment of localized disease and adjuvant mitotane is suggested for patients with high risk of recurrence. In the advanced setting, mitotane achieved an overall response rate (ORR) between 13% and 31%.^6^ In Fassnacht et al.‘s phase III trial, the frontline regimen of etoposide, doxorubicin, cisplatin (EDP), and mitotane had an ORR of 23.2% (with progression-free survival of 5 months) compared to 9.2% with streptomycin-mitotane, highlighting the dismal clinical outcomes.^7^

ACC is prone to histologic and phenotypic heterogeneity that creates challenges in diagnosis as well as creation of targeted therapies for treatment.^8^ While tissue-based biopsies have been the standard for interrogating genomic information, the availability of sufficient tissue and the need for repeat biopsies are common challenges to the tissue-based approach, especially in ACC. Blood-based circulating tumor DNA (ctDNA) testing is a viable approach that is minimally invasive, cost-effective, and able to dynamically characterize the genomic landscape in each patient, especially those who are medically unfit to undergo an invasive tissue biopsy. Multiple studies have demonstrated the concordance of ctDNA with tissue-based genomic assays in lung, prostate, colorectal, breast cancer, and other cancers.^9-13^ While there have only been a handful of studies documenting ctDNA’s applicability to ACC, their sample size is limited by the rarity of the disease as well as suboptimal ctDNA concentration for analysis.^14,15^ Developing a comprehensive understanding of ACC’s genomic landscape using ctDNA is an unmet need as it may pave the way to incorporating target-based therapies. Here we present the largest study to date evaluating the feasibility of a blood-based ctDNA approach in revealing clinically significant alterations and co-existing genomic alterations in ACC.

## Materials and Methods

We performed a de-identified, retrospective analysis of 120 patients with advanced ACC who have undergone plasma-based ctDNA next-generation sequencing (NGS) by Guardant360, a commercially available assay (Guardant Health, Redwood City, CA) between 2016 and 2021. Guardant360 is a Clinical Laboratory Improvement Amendments (CLIA)- and College of American Pathologists (CAP)-certified assay with high sensitivity and specificity for detecting cancer-related gene mutations.^16^ Our analysis interrogated single nucleotide variants, fusions, small insertions and deletions (indels), and copy number variations in up to 83 genes. Moreover, the frequency of genomic alterations, landscape of coexistent mutations, and frequency of pathogenic or likely pathogenic alterations was categorized. Pathogenic and likely pathogenic alterations with the potential to be sensitive to approved and/or investigational targeted therapies were characterized using OncoKB database, and we included levels 1, 2, and 3 as clinically relevant biomarkers.^17^ If patients had samples analyzed multiple times, their mutations and co-existing alterations were recorded only once to preclude duplications. The frequency of mutations detected in ctDNA was compared to the frequency of those identified in tissue NGS utilizing the publicly available cBioPortal database.^18,19^ These data were collected in accordance with Emory University Institutional Board Review Guidelines. Data transfer of de-identified results between Guardant Health and our institution was secured.

## Results

### Patient Demographics

We retrospectively evaluated genomic data from 120 patients with ACC who had ctDNA testing between 12/2016 and 10/2021 using a commercially available plasma-based NGS assay. The median age was 53 years (range, 21-81 years) and the majority of patients were female (56%; Table 1). Fourteen patients had ctDNA testing performed twice, and one patient had testing performed three times. Plasma-based NGS did not detect ctDNA in 24 (20%) patients. Among the entire cohort, 96 patients (80%) had at least 1 somatic alteration detected with a total of 47% (*n* = 56 patients) identified with pathogenic and/or likely pathogenic mutations in therapeutically relevant alterations. Of the potentially actionable alterations, 53 copy number amplifications were identified, 11 missense mutations, 7 indels, 4 splice site alterations, and 3 nonsense mutations (Table 2).

**Table 1.** Demographics and characteristics of patients with ACC who underwent circulating tumor DNA (ctDNA) testing.

	*N* = 120
Somatic mutations detected	80% (*N* = 96)
Female	56%
Age median, years	53
Age range, years	21-81
Two serial samples	14
Three serial samples	1

Abbreviations: ACC, adrenocortical carcinoma; ctDNA, circulating tumor DNA.

**Table 2.** Potentially actionable alterations detected in ACC cohort using ctDNA.

	Amp	Indel	Missense	Nonsense	Splice
*EGFR*	12	—	—	—	—
*BRAF*	7	—	2	—	—
*MET*	7	1	1	—	—
*CDK4*	6	—	—	—	—
*CDK6*	5	—	—	—	—
*ATM*	—	2	1		2
*CDKN2A*	—	1	2	1	—
*PDGFRA*	4	—	—	—	—
*KIT*	4	—	—	—	—
*PTEN*	—	1	2	1	—
*FGFR1*	3	—	—	—	—
*PIK3CA*	2	—	1	—	—
*CHEK2*	—	—	—	—	2
*FGFR2*	2	—	—	—	—
*BRCA1/BRCA2*	—	2	—	—	—
*CCND2*	1	—	1	—	—
*IDH2*	—	—	1	—	—
*MSH2*	—	—	—	1	—

Abbreviations: ACC, adrenocortical carcinoma; ctDNA, circulating tumor DNA.

### Molecular Alterations

**Figure 1.**
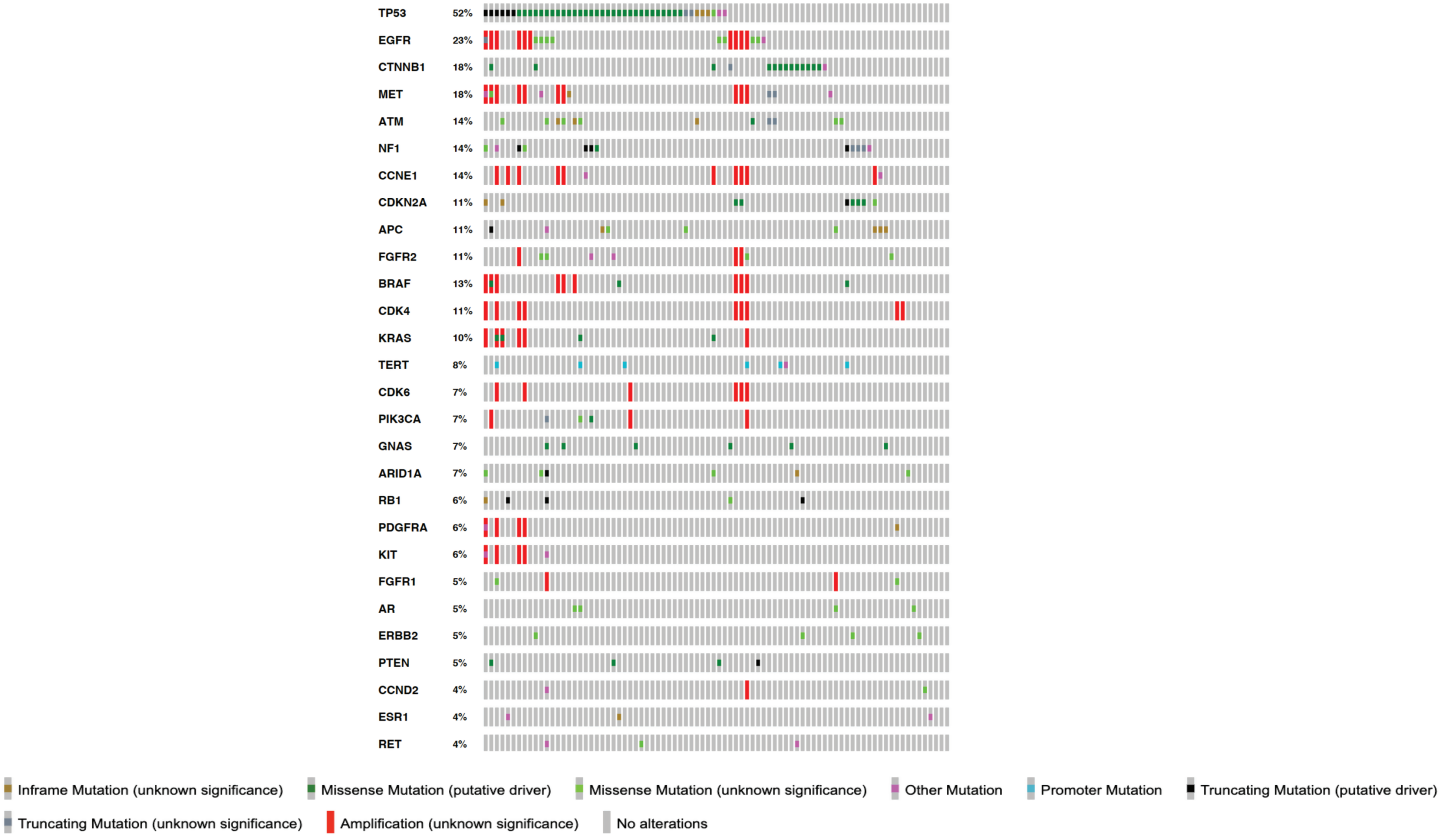
The most frequently detected genomic alterations in ACC patients, regardless of therapeutic implications. ACC, adrenocortical carcinoma.

Figure 1 reveals the most frequently mutated genes in our cohort, regardless of therapeutic relevance, of which *TP53* (52%), *EGFR* (23%), *CTNNB1* (18%), *MET* (18%), and *ATM* (14%) were the most common. Among the entire cohort, 56 patients (47%) had pathogenic and/or likely pathogenic mutations in therapeutically relevant alterations with existing therapies approved for other malignancies (Tables 2-4) with the most frequently detected mutations occurring in *EGFR* (13.5%), *BRAF* (12.5%), *MET* (10.4%), *CDK4* (7.3%), *CDKN2A* (7.3%), *ATM* (6.3%), and *CDK6* (6.3%).

**Table 3.** ctDNA mutations in therapeutically relevant alterations among patients with ACC.

Gene	*N*	%
*EGFR*	13	13.5
*BRAF*	12	12.5
*MET*	10	10.4
*CDK4*	7	7.3
*CDKN2A*	7	7.3
*ATM*	6	6.3
*CDK6*	6	6.3
*PTEN*	4	4.2
*PDGFRA*	4	4.2
*KIT*	4	4.2
*PIK3CA*	3	3.1
*FGFR2*	3	3.1
*FGFR1*	3	3.1
*CCND2*	2	2.1
*CHEK2*	2	2.1
*BRCA1/BRCA2*	2	2.1
*IDH2*	1	1.0
*MSH2*	1	1.0

Abbreviations: ACC, adrenocortical carcinoma; ctDNA, circulating tumor DNA.

**Table 4.** Drug approvals in other cancers for the therapeutically relevant genes detected in ACC cohort using ctDNA.

Gene	Therapeutic approvals in non-ACC patients	No. of patients with ACC
*EGFR*	afatinib, cetuximab, erlotinib, gefitinib, neratinib, panitumumab	13
*BRAF*	binimetinib, cobimetinib, dabrafenib, encorafenib, trametinib, vemerafenib	12
*MET*	cabozantinib, capmatinib, crizotinib	10
*CDK4*	abecmaciclib, palbociclib, ribociclib	7
*CDKN2A*	abecmaciclib, palbociclib, ribociclib	7
*ATM*	niraparib, olaparib, rucaparib, talazoparib	6
*CDK6*	abecmaciclib, palbociclib, ribociclib	6
*PTEN*	copanlisib, everolimus, temsirolimus	4
*PDGFRA*	dasatinib, imatinib, lenvatinib, nilotinib, nintedanib, olaratumab, pazopanib, ponatinib, regorafenib, sorafenib, sunitinib	4
*KIT*	axitinib, cabozantinib, dasatinib, imatinib, lenvatinib, nilotinib, pazopanib, ponatinib, regorafenib, sorafenib, sunitinib	4
*PIK3CA*	alpelisib, copanlisib	3
*FGFR2*	lenvatinib, nintedanib, pazopanib, ponatinib	3
*FGFR1*	erdafitinib, lenvatinib, nintedanib, pazopanib, pemigatinib, ponatinib	3
*CCND2*	abemaciclib, palbociclib, ribociclib	2
*CHEK2*	niraparib, olaparib, rucaparib, talazoparib	2
*BRCA1/BRCA2*	niraparib, olaparib, rucaparib	2
*IDH2*	enasidenib	1
*MSH2*	atezolizumab, avelumab, durvalumab, nivolumab, pembrolizumab	1

Abbreviations: ACC, adrenocortical carcinoma; ctDNA, circulating tumor DNA.

### Co-occurring Mutations

All co-occurring mutations were identified, and their statistical significance were determined and presented in Table 5.  The most common co-occurring mutations were *EGFR* + *MET* (*n* = 9), *MET + CDK4* (*n* = 7), *EGFR + CDK4* (*n* = 7), and *BRAF + MET* (*n* = 7). Other less common mutations include *MET + CDK6* (*n* = 5), *KRAS + CDK4* (*n* = 5), *CDK6 + CDK4* (*n* = 5), *CDK4 + PDGFRA* (*n* = 5), *CCNE1 + CDK4* (*n* = 5), *KRAS + PDGFRA* (*n* = 4), *KIT + PDGFRA* (*n* = 4), *KIT + KRAS* (*n* = 4), and *KIT + CDK4* (*n* = 4).

**Table 5.** Most-common co-occurring mutations detected in ACC cohort using ctDNA.

A	B	A and B Co-occurring	*P*	*Q*	Tendency
*MET*	*EGRF*	9	<.001	.007	Co-occurence
*MET*	*CDK4*	7	<.001	.001	Co-occurence
*EGFR*	*CDK4*	7	<.001	.016	Co-occurence
*BRAF*	*MET*	7	<.001	.007	Co-occurence
*MET*	*CDK6*	5	<.001	.012	Co-occurence
*KRAS*	*CDK4*	5	<.001	.016	Co-occurence
*CDK6*	*CDK4*	5	<.001	.003	Co-occurence
*CDK4*	*PDGFRA*	5	<.001	.001	Co-occurence
*CCNE1*	*CDK4*	5	<.001	.029	Co-occurence
*KRAS*	*PDGFRA*	4	<.001	.016	Co-occurrence
*KIT*	*PDGFRA*	4	<.001	.005	Co-occurence
*KIT*	*KRAS*	4	<.001	.035	Co-occurence
*KIT*	*CDK4*	4	<.001	.035	Co-occurrence

### Comparison of the Genomic Landscape in Blood ctDNA and Tissue-Based Sampling

**Figure 2.**
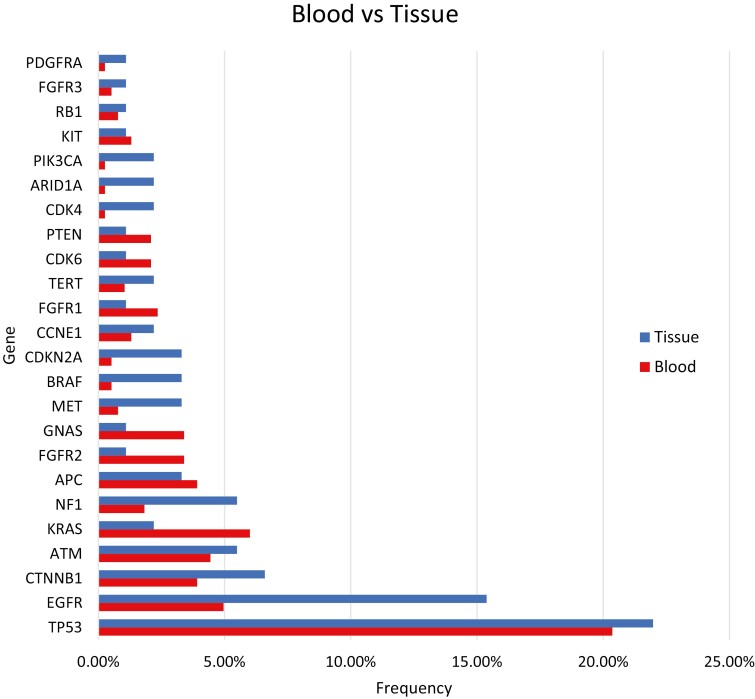
Comparison of detected genomic mutations in ACC using blood-based ctDNA (Guardant database) and tissue (cBioPortal). ACC, adrenocortical carcinoma; ctDNA, circulating tumor DNA.

We compared the mutation types and frequencies across the genomic landscape identified via ctDNA and tissue-based testing derived from cBioPortal. As seen in Figure 2, both the types and frequencies of mutations detected in potential activating drivers were similar across ctDNA and tissue-based testing. Of note, *KRAS* (6% vs. 2.2%), *BRAF* (3.3% vs. 0.5%), *APC* (3.9% vs. 3.3%)*, FGFR2* (3.4% vs. 1.1%), and *GNAS* (3.4% vs. 1.1%) were detected in ctDNA at a higher frequency than tissue-based sampling, respectively.

## Discussion

ACC is a rare and aggressive malignancy with poor prognosis in the advanced setting. The frontline standard of care chemotherapy drug regimen of EDP plus mitotane is characterized by short-lived responses and high incidence of toxicity. While ctDNA has been widely adopted and included in National Comprehensive Cancer Network guidelines for other tumor types, the characterization and use of ctDNA in advanced ACC have been limited. To our knowledge, this is the largest dataset to date and showed that ctDNA testing is feasible in advanced ACC, with 80% of tested patients having detected somatic alterations in the blood and close to half harboring pathogenic and/or likely pathogenic mutations in therapeutically relevant genes. The landscape of alterations was similar in plasma-based ctDNA and tissue-based testing. Mutations most frequently detected were in *EGFR*, *BRAF*, *MET*, *CDKN2A*, *CDK4/6*, and *ATM*. Moreover, 47% of these mutations were found to potentially be actionable with existing therapies that are approved in other cancer types.

The tissue-based genomic landscape of ACC in primary tumors has previously been characterized. Assie et al.,^20^ Lippert et al.,^21^ and Close et al.^22^ had provided an outlook into ACC’s genomic landscape with an emphasis on *TP53*, *CTNNB1*, *NF1*, *BRCA1/2* as the predominant mutations. Zheng et al.^23^ also highlighted *TP53*, *CTNNB1*, and *CCNE1* as potential driver mutations. Ross et al.^24^ conducted a similar study involving 29 patients and identified similar therapeutically relevant mutations (eg, *TP53*, *NF1*, *CDKN2A*, *MEN1*, *CTNNB1*, and *ATM*) and reported that approximately 60% of mutated genes in ACC could be targeted with therapies approved in other cancers. Furthermore, Crona et al. proposed three molecular subtypes of ACC based on chromosomal changes with prognostic implications and showed that close to 50% of patients with metastatic ACC have genetic alterations with approved therapies in other cancers.^25^ In our analysis, ctDNA NGS identified the same subset of mutations at a similar frequency to tissue-based sampling, not only highlighting its feasibility in concordantly revealing clinically relevant genetic alterations, but also uncovering mutations that may be key drivers in ACC pathogenesis.

In vitro investigations looked at targetable molecular pathways, and early phase I and II studies have delved into nucleotide- and genetic-based markers in advanced ACC in hopes of uncovering sensitivity to small molecule-based therapies. For instance, Voltante et al.^26^ discussed that low mRNA gene expression of ribonucleotide reductase large subunit correlated with better disease-free survival when treated with adjuvant mitotane. Other investigators showed that Mitotane-EDP might be more beneficial in patients with topoisomerase-2 alpha and excision repair cross-complementing group (*ERCC1*) mutations.^27,28^ This suggests that a biomarker-based treatment approach in ACC could be feasible.

The ACC treatment landscape has several emerging therapies, though no current approvals for a genomically targeted drug agent. In a retrospective cohort study of 16 patients with ACC previously treated with mitotane, subsequent treatment with cabozantinib, a multi-kinase inhibitor, had an encouraging disease control rate of 50%, with *N* = 3 partial responders and *N* = 5 with stable disease, along with a favorable safety profile.^29^ Furthermore, a phase II trial of pembrolizumab reported a 23% ORR in 39 patients with advanced ACC for any line of therapy, showing potential for anti-PD-1 or anti-PD-L1 based regimens.^30^ This suggests that ACC may have some sensitivity to immune-checkpoint inhibition (ICI), yet it remains unclear how the upregulated steroidogenesis pathways of ACC may impact response.^31,32^ Studies are currently investigating biomarkers of ICI response such as PD-1/L1 levels, microsatellite instability (MSI), and tumor mutational burden (TMB). Several case reports have demonstrated long-lasting therapeutic benefits with ICI in MSI-high patients with ACC,^33,34^ highlighting the importance of comprehensive genomic testing.

There are several advantages of ctDNA-based NGS over tissue-based, including the ease of repeat sampling to measure the changes in ctDNA, which are reflective of clinical response to therapy.^35,36^ This technique provides a faster turn-around time than tissue-NGS and can facilitate the enrollment of patients into molecularly selected clinical. Further, ctDNA could also be a tool to enhance detection of residual or recurrent disease in localized ACC.^15^

Our study had several limitations. First, there was no treatment or clinical information available for the de-identified patients. Thus, it is likely that the genomic landscape we identified was influenced by prior therapy, and more clinical information would have been needed to assess the representativeness of our findings. Second, our patient’s ctDNA results were not compared to their paired tissue-based sampling (a preferred approach when feasible), but rather an online public database. Lastly, there was no histology data available for our patients. Despite these limitations, the study has several strengths. It is the largest ctDNA analysis to date in rare tumor type, and all blood samples were tested at the same laboratory. Further, the results represent proof of concept that ctDNA testing is feasible and clinically relevant in advanced ACC.

## Conclusion

ACC is a rare and complex malignancy with poor prognosis and limited approved therapeutics beyond frontline mitotane-EDP chemotherapy. Identifying alterations with therapeutic implications using ctDNA could facilitate enrollment of patients into personalized therapy clinical trials. This non-invasive approach is feasible, provides similar results to tissue-based testing, and can inform the development of personalized targeted treatment options for this aggressive malignancy.

## Data Availability

The data underlying this article cannot be shared publicly due to privacy of individuals that participated in the study. The data summary can be shared on reasonable request to the corresponding author.
